# Anatomically remote muscle contraction facilitates patellar tendon reflex reinforcement while mental activity does not: a within-participants experimental trial

**DOI:** 10.1186/2045-709X-20-29

**Published:** 2012-09-07

**Authors:** Steven R Passmore, Paul A Bruno

**Affiliations:** 1 School of Medical Rehabilitation, Faculty of Medicine, University of Manitoba, R106 – 771 McDermot Avenue, Winnipeg, Manitoba, R3E 0T6, Canada; 2 Research Department, New York Chiropractic College, 2360 State Route 89, Seneca Falls, NY, 13148, USA; 3 Faculty of Kinesiology and Health Studies, University of Regina, 3737 Wascana Parkway, Regina, Saskatchewan, S4S 0A2, Canada

**Keywords:** Neurologic examination, Stretch reflex, Jendrassik maneuver, Stroop task, Presynaptic inhibition

## Abstract

**Background:**

The Jendrassik maneuver (JM) is a remote facilitation muscular contraction shown to affect amplitude and temporal components of the human stretch reflex. Conflicting theoretical models exist regarding the neurological mechanism related to its ability to reinforce reflex parameters. One mechanism involves the gamma motoneurons of the fusimotor system, which are subject to both physical and mental activity. A second mechanism describes reduced alpha motoneuron presynaptic inhibition, which is not subject to mental activity. In the current study, we determined if mental activity could be used to create a reflex facilitation comparable to a remote muscle contraction.

**Method:**

Using a within-participants design, we investigated the relative effect of the JM and a successfully employed mental task (Stroop task) on the amplitude and temporal components of the patellar tendon reflex.

**Results:**

We found that the addition of mental activity had no influence on the patellar tendon reflex parameters measured, while the JM provided facilitation (increased reflex amplitude, decreased total reflex time).

**Conclusion:**

The findings from this study support the view that the mechanism for the JM is a reduction in presynaptic inhibition of alpha motoneurons as it is influenced by physical and not mental activity.

## Introduction

If a tendon reflex is not elicited during a neurological examination, a clinician may reattempt the procedure with reinforcement. One such reflex reinforcement technique is the Jendrassik maneuver (JM), which employs a voluntary anatomically remote muscle contraction concurrent with reflex elicitation [1]. The JM’s effect on amplitude and temporal components of the patellar tendon reflex has been demonstrated [2-8]. The underlying neurological mechanism of the JM remains elusive and is a source of debate.

Two predominant but conflicting theories related to two different types of motoneurons have been proposed, and are feasible for scholarly discussion. One theory suggests that the JM acts via fusimotor activation. The fusimotor or gamma system consists of gamma motoneurons acting in response to changes detected in muscle spindle activity from Ia afferent fibres. The fusimotor system activity can be modulated by changes in the level of activity in either the mental, or physical state [9]. This was tested by comparison of microneurographic studies measuring muscle spindles in the tibialis anterior (TA) in a resting state compared to states of mental and physical action. They found that an enhanced sensitivity to stretch of muscle spindles compared to a rest condition occurred following either mental computation or voluntary fist clenching (which is an anatomically remote muscle contraction from the TA creating a JM). A second theory used to explain the JM is a reduction in presynaptic inhibition (PSI) of alpha motoneurons by Ia afferents. Hultborn, Meunier, Pierrot-Deseilligny, and Shindo [10] found that a focused muscle contraction decreases PSI. Zehr and Stein [11] presented a background electrical stimulation of the common peroneal nerve, which created a 10% contraction of the soleus muscle (increased PSI) intended to create reflex inhibition. On separate trials that utilized a JM by asking participants to clench their teeth, and pull their interlocked fingers away from each other, a context for reflex facilitation was created. A linear summation/cancellation effect was noted when both an induced background contraction, and a JM were utilized in tandem. Zehr and Stein [11] determined that the equilibrium between factors that increase or decrease PSI may serve as the mechanism for the JM. Both theories have been challenged [12], but not completely refuted, particularly since the fusimotor system can operate independently of the alpha motor system [9]. A third theory that the JM is a direct facilitation of alpha motoneurons has been largely dismissed [13].

A Stroop task (ST) is considered the gold standard measure of attention [14]. The traditional Stroop colour naming paradigm requires the participant to identify either the word presented (which is a colour), or the colour of the font the word is presented in [15]. The potential for interference arises when the semantic meaning of the word differs from the colour of the font and the participant must make a response decision. For example, if the word “BLUE” was presented in a red font, such a context is incongruent. It is a cognitive task that requires a participant to utilize both their working memory, and endogenous attentional focus in order to respond correctly [16]. A ST is commonly employed as a secondary task added to a primary motor task condition, creating a dual task situation. The dual task condition frequently results in a decrement in performance of the primary task [17,18]. For example, performing a ST while attempting to execute a rapid voluntary step in response to a tap cue on the heel (dual task condition) led to longer step initiation times in younger and older adults compared to executing the step response alone (primary task condition) [17]. Performing a ST while walking on a level surface (dual task condition) led to changes in the balance control strategies of younger and older adults (e.g. decreased walking velocity, decreased step length) compared to walking alone (primary task condition) [18].

In the current study, we determined if mental activity could be used to create a reflex facilitation comparable to a voluntary remote muscle contraction (JM). Specifically, we employed a mental task of sufficient complexity to capture attention that also required a cognitive decision (Stroop task). If no difference was found between the mental task and the traditional motoric JM task on reflex parameters, but both created reflex facilitation compared to a rest condition, it would indicate that fusimotor activation was a probable mechanism for the JM. The mechanism would be indicated since fusimotor activation is known to be impacted by both mental and physical activity. In contrast, if only the JM condition enhanced reflex parameters, support would be provided for the theory that the JM is a reduction in PSI of alpha motoneurons by Ia afferents, which is influenced by physical and not mental activity.

## Method

### Participants

In a within-participants design, 18 healthy adult volunteers (13 females) with a mean age of 29.2 years (*SD* = 4.2) were recruited for the study. All participants were naïve to the purpose of the study. A priori exclusion criteria included: adults over 35 years of age, pregnancy, history of debilitating injury within the previous three months, history of injury or surgery to the right knee, history of a spinal arthritic disorder, history of lumbar nerve root compression (e.g. intervertebral disc herniation), and history of a central/peripheral nervous system or neuromuscular disorder. Two participants were later excluded, one for colour-blindness and another who misunderstood the ST instructions, leaving 16 participants for final analysis. All participants provided written informed consent. The study was approved by the University of Regina Research Ethics Board.

### Procedures

Participants were seated with their hips and knees in 90° of flexion, at a height where their feet could not touch the ground [3,5,6,19]. A series of taps was delivered to the right patellar tendon. Two blocks of 10 taps (20 total taps) [20] were elicited for each of the following conditions: (1) *Rest* – participants were asked to sit as relaxed as possible; (2) *JM* – participants attempted to pull their interlocked fingers apart with maximum effort [3,8]; and (3) *ST* – participants performed a block of 12 Stroop colour-word test trials on a laptop monitor [14,15]. The condition order was randomly assigned for each participant to control for order effects and possible habituation in reflex responses over time. To maximize comfort and minimize muscle fatigue, 2–3 minute rest periods occurred between blocks. To minimize anticipatory responses, variable inter-tap intervals of 10–20 seconds were used [21].

Each block of ST trials consisted of 4 neutral, 4 congruent, and 4 incongruent scenarios, which were presented in a random order. At a random point during each block of 12 trials, a single tap was delivered to the patellar tendon. Participants placed their middle and index fingers on keyboard letters *q*, *e*, *i*, and *p*. The keyboard letters had coloured stickers indicating *red*, *green*, *orange*, and *blue*, respectively. Participants were asked to respond to the colour of the font presented as quickly and accurately as possible. Neutral trials required a response to the letters *XXXX*. In the congruent trials, the word and colour font presented were the same (e.g. *BLUE* in a blue font). In the incongruent trials, the word and colour presented were not the same (e.g. *BLUE* in an orange font). For each Stroop trial a fixation symbol (*#*) was presented (250 ms) in the middle of the screen, followed by a post-fixation interval (900 ms) and stimulus presentation (100 ms). An infinite duration was allowed for the participant to respond. After selecting their response, an inter-trial blank screen interval was presented (1000 ms) before the next trial began. To familiarize participants with the task, each participant completed 2–3 practice blocks of ST trials that were performed without concurrent reflex delivery. All ST trial presentations and response recordings were performed using custom E-prime 2.0 software (Psychology Software Tools Inc., Sharpsburg, PA).

### Experimental apparatus and equipment

**Figure 1:**
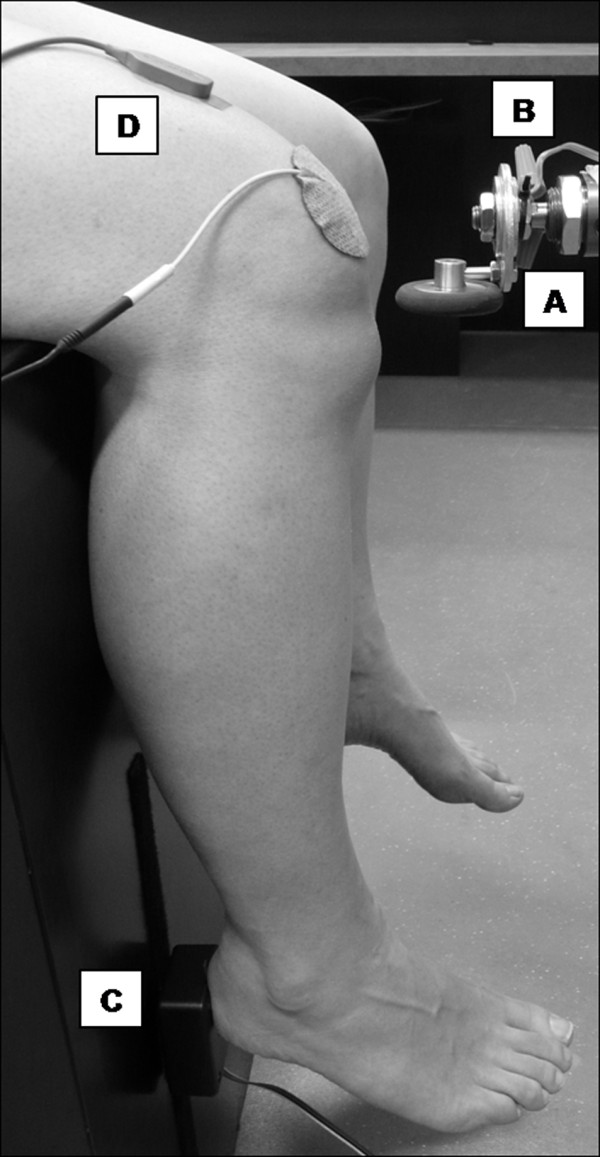
**Participant position and experimental apparatus.A**) Custom-built pneumatic device that delivered the patellar tendon taps. **B**) Mercury switch mounted to the tapper. **C**) Microswitch mounted behind the participant’s heel. **D**) Surface electromyography sensor.

A custom-built pneumatic tendon tapper (Figure 1A) was used to keep the amplitude, pressure, and location of the taps constant due to a demonstrated relationship between stimulus strength and reflex amplitude [22,23]. The device consisted of a reflex hammer head mounted on the tip of a pneumatic piston driven by an air compressor. The compressor maintained a tapping pressure of 75 psi and was triggered by a manual switch. The person who triggered the manual switch stood behind the participant to prevent him/her from detecting visual cues related to switch activation. The tapper system was attached to a stand with adjustable height, allowing the hammer to be set at an appropriate level for each participant. A mercury switch mounted on the hammer head (Figure 1B) was triggered when the piston reached its end point (i.e. its impact with the patellar tendon). A microswitch mounted behind the participant’s heel (Figure 1C) recorded the participant’s reflex movement response onset (i.e. the heel leaving the switch). Switch onsets were recorded with millisecond precision using custom E-prime 2.0 software (Psychology Software Tools Inc., Sharpsburg, PA). 

The skin over the participant’s right distal thigh and right patella was shaved (if necessary) and swabbed with alcohol. A DE-2.3 Single Differential Surface Electroymyography (EMG) Sensor (DelSys Inc., Boston, MA) was attached to the skin in the midline of the thigh 5 cm proximal to the superior patellar margin (Figure 1D). The sensor’s two parallel Ag bars (10 mm in length, 1 mm in diameter, spaced 10 mm apart) were oriented perpendicular to the underlying rectus femoris muscle fibres. A reference electrode was attached to the skin over the patella. During each repetition, raw surface EMG activity was detected, amplified (gain: 1000), bandpass filtered (20–450 Hz), A-D converted (sampling rate: 2000 Hz), and recorded using a Myomonitor IV EMG System (DelSys Inc., Boston, MA).

A series of 5–10 practice taps were performed to ensure that the pressure level achieved consistent reflex responses while ensuring participant comfort.

### Dependent variables

Dependent variables for the three conditions (rest, JM, ST) included total reflex time (TRT) and reflex amplitude. TRT was the time (ms) difference between the onsets of the two switches (i.e. between the impact of the hammer head and the onset of heel movement) for each tap [3,5,6,19]. Reflex amplitude was the peak-to-peak amplitude (μV) of the EMG response to each tap [8,22-25]. This was calculated for each repetition with EMGworks Analysis 4.0 software (DelSys Inc., Boston, MA). To minimize bias, the investigator who performed the EMG data analysis was blinded to the condition of the set being analyzed.

Additional dependant variables for ST trials included reaction time and response accuracy. Reaction time was the time (ms) difference between the word stimulus presentation and the response initiation. Response accuracy was the percent correct in each ST category (neutral, congruent, incongruent).

### Statistical analysis

All recorded dependent variables were measured on interval, and not nominal or ordinal scales. As a result all analyses were conducted using parametric statistical testing. For each participant, the truncated mean TRT, reflex amplitude, and reaction time (ST trials) were calculated for each condition/category. Truncation involved removal of any outlier data points beyond 2 *SD* from the mean calculated for the total number of repetitions/trials performed for the condition/category. Means were compared across conditions via one-way repeated measures ANOVA models with a 1(participant) x 3(condition/category) design using SPSS 15.0 software (LEAD Technologies Inc, Charlotte, NC). Greenhouse-Geisser corrected degrees of freedom were used in cases of violated sphericity with corrected degrees of freedom and appropriate epsilon values reported. Post-hoc analyses were performed using Bonferroni pairwise comparisons, followed by Cohen’s *d* to measure effect sizes.

## Results

### Tendon reflex parameters

**Figure 2:**
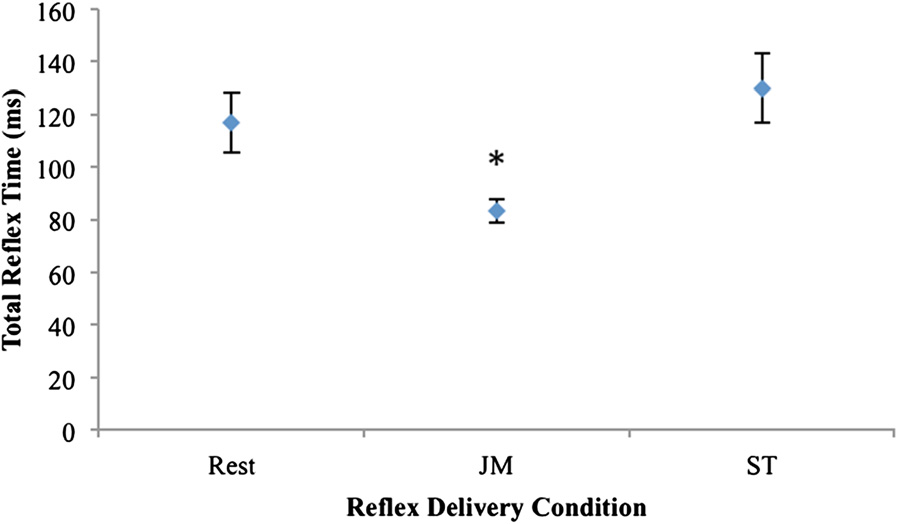
**Mean ±**  ***SE *(error bars) total reflex times of the three conditions (n = 16). ** Asterisk denotes statistically significant differences between Jendrassik maneuver (JM) and rest, and JM and Stroop task (ST).

For TRT, a significant main effect was found for condition, *F*(2,30) = 14.356, *η*_*p*_^*2*^ = .489, *p* < .001. Pair-wise comparison revealed significant differences between JM and rest conditions (*d* = .976), and JM and ST conditions (*d* = 1.182) (Figure 2 and Table 1).

**Table 1:** Mean total reflex times and reflex amplitudes of the three conditions (n = 16)

	**Total reflex time (ms)**	**Reflex amplitude (μV)**
**Condition**	***M (SE)***	***M (SE)***
Rest	117.1 (11.5)	256.0 (77.7)
JM	83.1 (4.5)*	500.7 (94.6)*
ST	130.1 (13.3)	265.9 (68.2)

*Statistically significant differences between Jendrassik maneuver (JM) and rest, and JM and Stroop task (ST).

**Figure 3:**
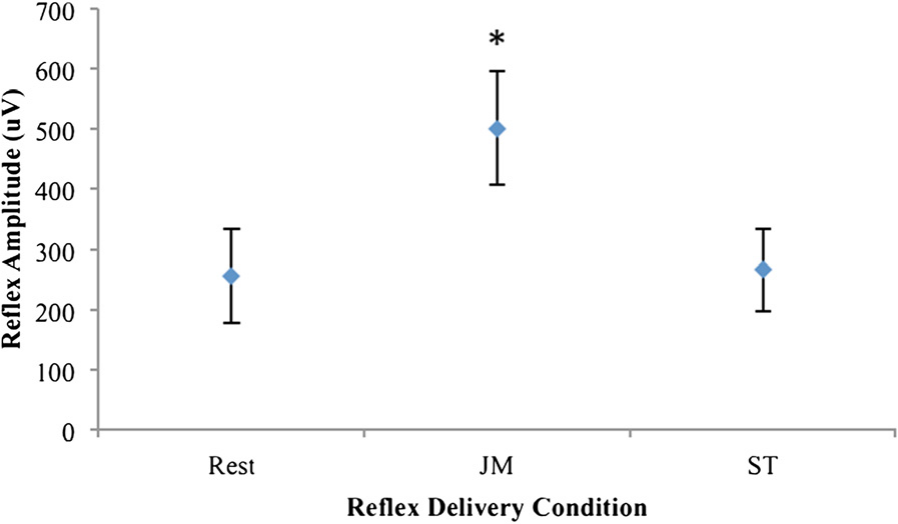
**Mean ±**  ***SE *(error bars) reflex amplitudes of the three conditions (n = 16). ** Asterisk denotes statistically significant differences between Jenrassik Maneuver (JM) and rest, and JM and Stroop task (ST).

For reflex amplitude, a significant main effect was found for condition, *F*(1.241,18.617) = 20.369 , *η*_*p*_^*2*^ = .576, *p* < .001, where ϵ = 0.621. Pair-wise comparison revealed significant differences between JM and rest conditions (*d* = .707), and JM and ST conditions (*d* = .712) (Figure 3 and Table 1).

### Stroop task (ST)

**Figure 4:**
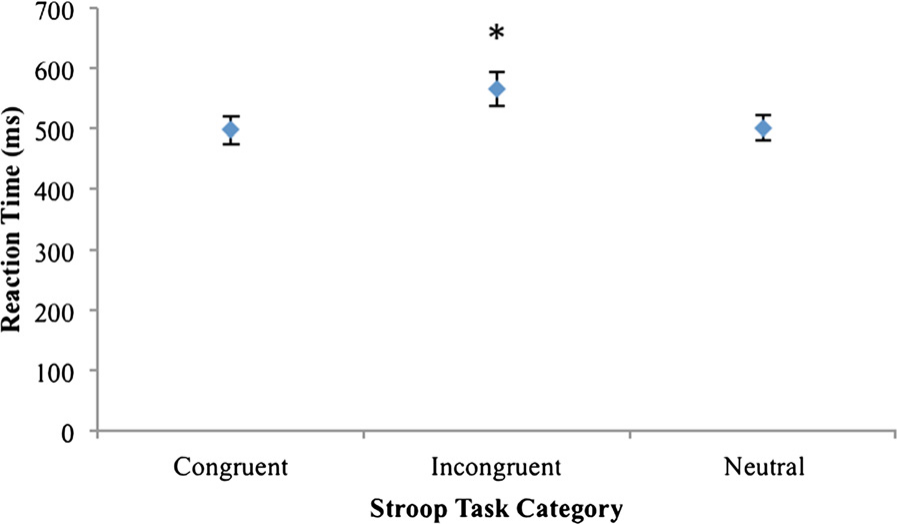
**Mean ± *SE *(error bars) reaction times of the three Stroop task categories (n = 16).** Asterisk denotes statistically significant differences between incongruent and neutral categories, and incongruent and congruent categories.

For reaction time, a significant main effect was found for task category, *F*(2,30) = 26.329, *η*_*p*_^*2*^ = .637, *p* < .001. Pair-wise comparison revealed significant differences between incongruent and neutral categories (*d* = .648), and incongruent and congruent categories (*d* = .660) (Figure 4 and Table 2). Regardless of category, all mean response accuracy scores exceeded 95% correct for each participant.

**Table 2:** Stroop task mean response accuracy and reaction time (n = 16)

	**Response accuracy (%)**	**Reaction time (ms)**
**Stroop task condition**	***M (SD)***	***M (SE)***
Congruent	95.9 (2.7)	497.9 (22.3)
Incongruent	97.6 (1.7)	565.8 (28.3)*
Neutral	95.4 (1.3)	500.9 (20.8)

*Statistically significant differences between incongruent and neutral, and incongruent and congruent.

## Discussion

Our finding that the JM significantly decreased the TRT and significantly increased the amplitude of the patellar tendon reflex (Figures 2 and 3) replicates previous results [3,5-8,22-25]. A direct comparison of previously-reported values for these outcomes is difficult due to the heterogeneity of methodology and apparatus between studies. While the use of electronic switches on the hammer head and behind the participant’s heel to define the TRT, and the peak-to-peak amplitude of the EMG response to the tendon tap to define the reflex amplitude, have been used in multiple studies (see 2.4 Dependent Variables), techniques incorporating the use of load cells [2,26], electrogoniometers [27], or accelerometers [28] have also been used to describe parameters of the patellar reflex. There are a sufficient number of studies with comparable methods to illustrate an informal comparison with our results. Such reported values for patellar tendon reflex TRT [3,5-7] and amplitude [7,8,22-25] in representative samples of asymptomatic individuals are provided in Table 3, and Table 4, respectively. 

**Table 3:** Previously reported mean patellar tendon reflex total reflex time and change scores

		**TRT**_**REST**_** (ms)**	**TRT**_**JM**_**(ms)**	**TRT Change**_**REST - JM**_** (%)**
**Study**	***n***	***M (SD)***	***M (SD)***	***M***
Clarkson (1978)	NR	83.1 (NR) to 86.3 (NR)	77.6 (NR) to 80.6 (NR)*	−5.7 to −7.6
Hayes (1972)	16	115.0 (26.8)	99.5 (NR)*	−13.5
Jacobson & Edwards (1990)	10	103.4 (15.4)	N/A	N/A
Kroll (1968)	25	70.8 (20.1)	58.8 (10.9)*	−16.9
Current study	16	117.1 (45.9)	83.1 (17.9)*	−29.0

Abbreviations: Total reflex time (TRT), Jendrassik maneuver (JM), not reported (NR), not applicable (N/A).

*Statistically significant difference between JM and rest.

**Table 4:** Previously reported mean patellar tendon reflex EMG amplitude and change scores

		**Amplitude **_**REST**_**(mV)**	**Amplitude **_**JM**_**(mV)**	**Amplitude Change **_**REST - JM**_**(%)**
**Study**	***n***	***M (SD)***	***M (SD)***	***M***
Frijns et al., (1997)	102	1.8 (1.2)	2.4 (1.4)*	37.3
Kuruoglu & Oh (1993)	24	1.4 (0.9)	N/A	N/A
Stam & Tan (1987)	15	1.2 (0.2)	N/A	N/A
Stam & van Creval (1989)	40	1.9 (NR)	N/A	N/A
Toulouse & Delwaide (1980)	20	NR	NR	180.0
Zabelis et al., (1998)	52	0.7 (0.5)	1.2 (1.7)*	80.6
Current study	16	0.26 (0.31)	0.50 (0.38)	95.6

Abbreviations: Jendrassik maneuver (JM), not reported (NR), not applicable (N/A).

*Statistically significant difference between JM and rest.

Our sample’s mean TRT during the rest and JM conditions (117.1 ms and 83.1 ms, Figure 2) appear comparable to those found in previous studies (Table 3). However, our mean percentage change between the conditions (−29.0%) appears somewhat higher than previous reports. Conversely, our sample’s mean reflex amplitude during the rest and JM conditions (256.0 μV and 500.7 μV, Figure 3) appears lower than those found in previous studies (Table 4), while our mean percentage change between the conditions (+95.6%) is within the range of previous reports. One possible explanation for these inconsistencies relates to slight differences in study methodologies. We will highlight those related to participant positioning, the specific facilitation contraction used, and EMG electrode placement.

Our participants were seated with their hips and knees in 90° of flexion (see Procedures). Although this mirrors the participant positioning used in several studies, many authors have utilized a supine position with the knee in 45° of flexion [22-25]. The impact of such a difference in participant positioning on the outcomes has not been reported. Similarly, although our choice of facilitation contraction (see Procedures) has been used in some studies, other types of remote contractions (e.g. teeth clenching) have been used to elicit the JM effect [5-7]. Finally, the choice of EMG electrode placement shows the most between-study variability, with some authors reporting the use of relatively large inter-electrode distances [22,23]. Such methods would have an effect on the resulting EMG signals. During pilot testing, several electrode placement options were compared with the site selected for our study (see Experimental apparatus and equipment) being that which consistently resulted in the least amount of baseline noise without minimizing the signals resulting from the tendon taps. The previous study whose selection of electrode placement was most similar to ours [8] also reported peak-to-peak amplitude values that most closely approximated our values.

Regardless of the methodological differences between studies, there is consistent support in the literature that the JM significantly decreases the TRT and significantly increases the amplitude of the patellar tendon reflex. In contrast, although our ST was successfully implemented and traditional Stroop findings were reproduced (Figure 4 and Table 2), performance of this task yielded no significant changes in reflex parameters compared to the rest condition (Figures 2 and 3).

In the present study we were able to discern that mental activity did not elicit a measurable effect on reflex amplitude, or TRT. Based on these findings, our results lend credence to the theory that the mechanism of the JM is not dependent on the gamma motoneurons of the fusimotor system, which is known to be influenced by mental activity. Instead, a theoretical framework that is supported by our findings is a mechanism that is not influenced by mental activity, but only by remote muscle contraction (a JM). Based on the current literature, that mechanism is reduced presynaptic inhibition of alpha motorneurons by Ia afferent fibres.

Support for this model is, however, not universal. Although our ST did not produce an appreciable effect on the TRT or amplitude of the patellar tendon reflex, mental activity in the form of motor imagery (i.e. mental simulation of a muscle contraction) has been shown to facilitate tendon reflex and/or H-reflex responses of the soleus muscle [29-31]. In addition, patients with radial nerve palsy who attempted to contract their ipsilateral wrist extensors (resulting in no contraction due to the palsy) demonstrated a small degree of patellar tendon reflex facilitation during the period of attempted contraction [4]. Experimental results presented by Gregory, Wood, and Proske [9] challenge both of the predominant theoretical models of JM facilitation in the literature (i.e. fusimotor activation, reduced presynaptic inhibition of alpha motorneurons), leading these authors to theorize that the JM “does not involve the motoneurones directly, but must act at an interneuronal site somewhere upstream”. It is beyond the scope of our study to comment further on the potential contribution of such “oligosynaptic pathways” [9].

A potential limitation of our study is that the JM’s and ST’s effect on the participants’ state of arousal is uncertain. Kobayashi, Yoshino, Takahashi, and Nomura [32] demonstrated that the ST influences a person’s state of arousal as measured using changes in skin conductance responses (SCR). McIntyre, Ring, and Carroll, [33] tested mental computation (a task requiring cognition), compared to number repetition (attentional capture task), and found that changes in cardiovascular arousal occured with the computation task only. Extension of their study to include a measure of arousal during a physical JM could add more clarity to the role of arousal in reflex modulation and if that mechanism differs from the JM. Stimuli capable of inducing anxiety, sexual, or cardiovascular arousal can impact spinal reflex parameters [33-35], but as of yet there is no evidence that a task that provokes arousal changes in SCR impacts spinal reflexes. Stimuli that induce arousal are predicted to be indicative of increased sympathetic nervous system activity, which is not thought to be related to the JM, and have been dismissed as being unrelated to the fusimotor system [9]. In a clinical context, it would not be practical to over-stimulate a participant’s sympathetic nervous system for the purpose of spinal reflex reinforcement.

## Conclusions

In summary, the findings of our study successfully replicated the traditional Jendrassik paradigm of reflex reinforcement. A classic Stroop effect was successfully replicated by our mental activity, yet it had no impact on TRT or reflex amplitude compared to the control condition. Thus, a task that was primarily mental activity-based in nature had no influence on the reflex parameters measured, while the traditional JM provided facilitation. The findings from this study provide support for the view that the mechanism for a JM is a reduction in PSI of alpha motoneurons by Ia afferents as it is influenced by physical and not mental activity. We conclude that clinicians who wish to reinforce a tendon reflex during a neurological examination would be advised to employ a method that is based on remote muscle contraction, rather than a concurrent mental activity.

## Abbreviations

JM: Jendrassik maneuver; TA: Tibialis anterior; PSI: Presynaptic inhibition; ST: Stroop task; EMG: Electroymyography; TRT: Total reflex time; ANOVA: Analysis of variance; SCR: Skin conductance responses.

## Competing interests

The authors declare that they have no competing interests.

## Authors’ contributions

Both authors contributed equally to all aspects of the experiment and manuscript.
